# A case of IgG4-related disease manifesting as a spinal epidural mass

**DOI:** 10.1093/bjrcr/uaae022

**Published:** 2024-07-04

**Authors:** Hatty Hoi Ting Chau, Bill Archie Lo, Wai Pong Chu, Ho Nam Ho, Wilson Man-shan Tsui

**Affiliations:** Radiology Department, Princess Margaret Hospital, Kowloon, Hong Kong SAR; Radiology Department, Tseung Kwan O Hospital, Kowloon, Hong Kong SAR; Department of Radiology, Northern Care Alliance National Health Service (NHS), Salford, M6 8HD, United Kingdom; Radiology Department, Tseung Kwan O Hospital, Kowloon, Hong Kong SAR; Department of Clinical Pathology, Tseung Kwan O Hospital, New Territories, Hong Kong SAR

**Keywords:** IgG4, IgG4-related disease, IgG4-related hypertrophic pachymeningitis, hypertrophic pachymeningitis, epidural mass, MRI

## Abstract

IgG4-related disease is an immune-mediated fibroinflammatory condition. Isolated manifestation in the spine as hypertrophic pachymeningitis is very rare and the mass-like lesion on MRI often mimic tumour or infection. Patients would present with symptoms that result from mass effect or neurovascular compression. Studies showed that serum and CSF IgG4 levels are rarely informative, and therefore, tissue biopsy is crucial for accurate diagnosis. Apart from supporting the diagnosis, MRI is helpful in delineating the extent of disease and follow-up after treatment. A 18F-FDG PET/CT scan is useful in detecting systemic manifestations of IgG4-related disease. Although IgG4-related disease generally responds well to corticosteroid at inflammatory state, relapse is not uncommon. Current treatment strategies for IgG4-related hypertrophic pachymeningitis are high dose corticosteroid therapy and early decompressive surgery to avoid chronic neurological complications. We described a case of a 27-year-old gentleman complaining of lower limb weakness and numbness. MRI showed a mass-like epidural lesion at the thoracic spine causing cord compression. Open biopsy of the epidural mass demonstrated histopathological characteristics of IgG4-related disease. Patient responded well to early surgical decompression of the spinal cord and corticosteroid as evidenced by symptom improvement and resolving mass on subsequent MRI study. However, a follow-up MRI revealed disease recurrence years later.

## Introduction

IgG4-related disease (IgG4-RD) is an immune-mediated fibroinflammatory condition featured by extensive IgG4-positive plasma cells and T-lymphocyte infiltration of various organs.^1^ It can involve multiple organs systematically or in isolation.^2^ IgG4-related hypertrophic pachymeningitis (IgG4-HP) is a rare form of IgG4-RD manifestation.^3^ Isolated IgG4-HP manifestation in spine is even more unusual.^3^ It can present as a mass-like lesion on MRI which often mimic tumour or infection. Patients would present with symptoms that result from mass effect or neurovascular compression.^4^ Here, we present a case of a 27-year-old male complaining of lower limb weakness and numbness. In this patient, an open biopsy of epidural mass at the thoracic spine demonstrated histopathological characteristics of IgG4-RD. Patient responded well to early surgical decompression of the spinal cord and higher dose corticosteroid at first presentation but disease recurrence happened years after when dosage of maintenance corticosteroid is reduced.

## Clinical presentation

A 27-year-old male with insidious onset of bilateral lower limb weakness and numbness over 3 weeks was assessed by orthopaedic surgeons. There was no history of fever, injury, neck pain, or back pain. Physical examination revealed mild tenderness over the spine at the mid thoracic level, reduced sensation below T10 level, reduced bilateral lower limb muscle power (grade 4 over 5). Hyperreflexia of bilateral knee and ankle jerks was elicited.

## Investigations and imaging findings

**Figure 1.**
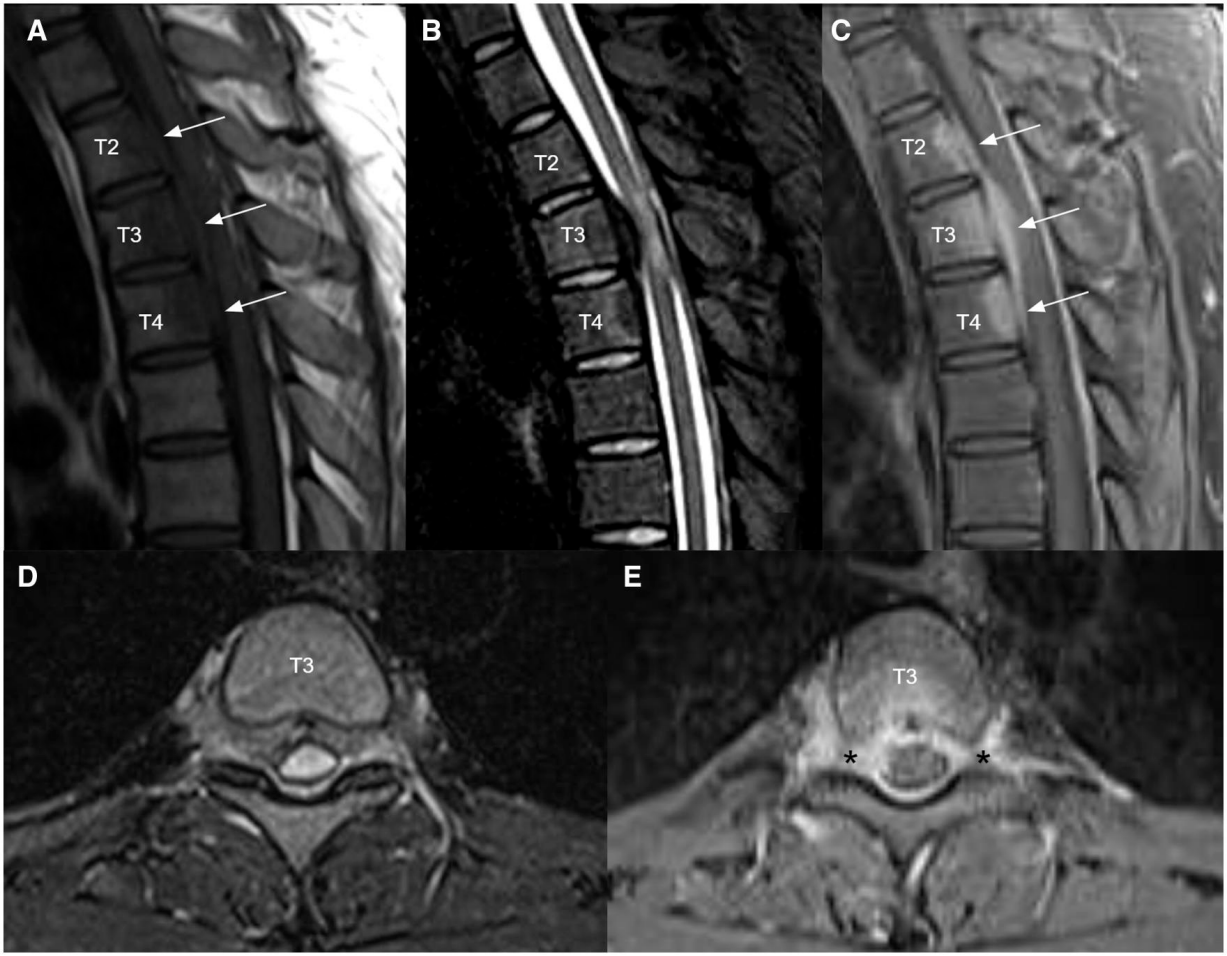
Sagittal T1 weighted (A), T2 STIR (B), and T1 weighted post-contrast (C) MRI images of upper thoracic spine demonstrated an enhancing infiltrative anterior epidural mass at T2 to T4 levels (white arrows) causing mild cord compression mainly at T3 level with cord oedema. Axial T2 STIR (D) and T1 weighted post-contrast (E) at T3 level showed that the epidural mass infiltrates into bilateral T3 lateral recesses and bilateral T3/4 intervertebral foramina (black asterisks). Enhancing lesions were also noted at the posterior aspect of T2 to T4 vertebral bodies (E).

**Figure 2.**
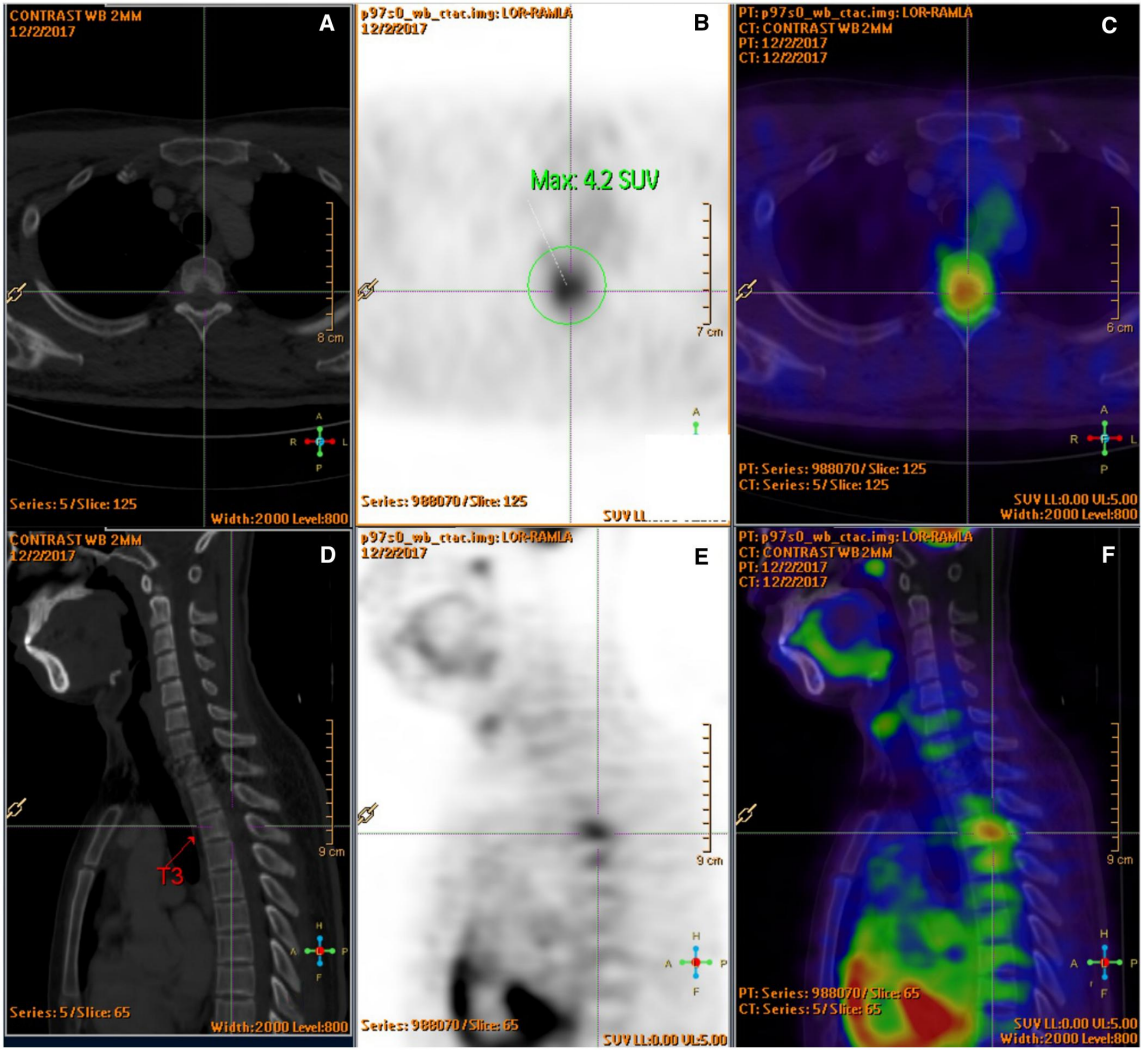
(A-F) F-18 FDG PET-CT showed an intraspinal mass at T3 level with increased FDG uptake (SUV max 4.2). No overt bone erosion or extraspinal hypermetabolic lesion was detected.

Blood tests including complete blood count, bone profile, erythrocyte sedimentation rate, C-reactive protein, rheumatoid factor, antinuclear antibodies, and tumour markers were normal. Thoracolumbar radiographs and CT brain were unremarkable. MRI of the thoracic spine (Figure 1) revealed a spindle-shaped anterior epidural mass at T2 to T4 levels causing mild cord compression mainly at T3 level with cord oedema (Figure 1A-C). The epidural mass infiltrated into bilateral T3 lateral recesses and bilateral T3/4 intervertebral foramina (Figure 1D and E). Enhancing lesions were also noted at the posterior aspect of T2 to T4 vertebral bodies (Figure 1C). 18F FDG PET/CT confirmed an intraspinal mass at T3 level with increased FDG uptake (SUV max4.2) (Figure 2). The scan also excluded intracranial involvement and involvement in other spinal levels. No overt bone erosion or extra-neurological hypermetabolic lesion was detected. Provisional imaging differential diagnosis included malignancy (eg, lymphoma, small round cell tumour), infection (eg, tuberculosis), and inflammation (eg, IgG4-RD).

## Treatment and tissue biopsy results

**Figure 3.**
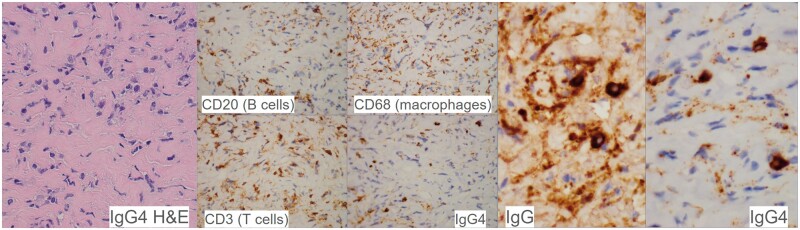
Histopathological findings in the epidural lesion. The lesion consists of fibrotic tissue with lymphoplasmacytic infiltrate between lamellated collagen fibres. The inflammatory cells comprise a mixture of B and T lymphocytes, plasma cells and macrophages. The plasma cells were mostly positive for IgG4, with an IgG4: IgG ratio >90%. Pathological diagnosis was IgG4-related fibro-inflammatory lesion.

Surgical exploration of the spinal canal with laminectomy was performed. Intraoperation found an intraspinal whitish fibrotic mass with dense adhesion to ventral dura at T2 to T4 level. Frozen section showed dense fibrous tissue with lymphoid infiltrate and atypical cells. Decompression of T3 spinal canal by partial excision of the fibrotic mass was done. Tissue biopsy (Figure 3) revealed fibrotic tissue with mononuclear inflammatory cells (B and T lymphocytes, plasma cells, and macrophages) between the collagen fibres. There were 20 IgG4+ plasma cells per high power field with an IgG4 to IgG ratio of more than 90%. Pathological diagnosis was IgG4-related fibro-inflammatory lesion. However, postoperative serum IgG4 was not elevated. Patient was being treated with moderate dose corticosteroid which gave a good response.

## Outcome and follow-up

Patient’s lower limb power and sensation progressively improved and symptoms resolved eventually. He stayed on low dose corticosteroid (oral prednisolone 10-7 mg daily) for 12 months. Subsequent follow-up MRI at 12 months showed resolution of the anterior epidural mass along T2-T4 level. There was no more spinal cord compression.

**Figure 4.**
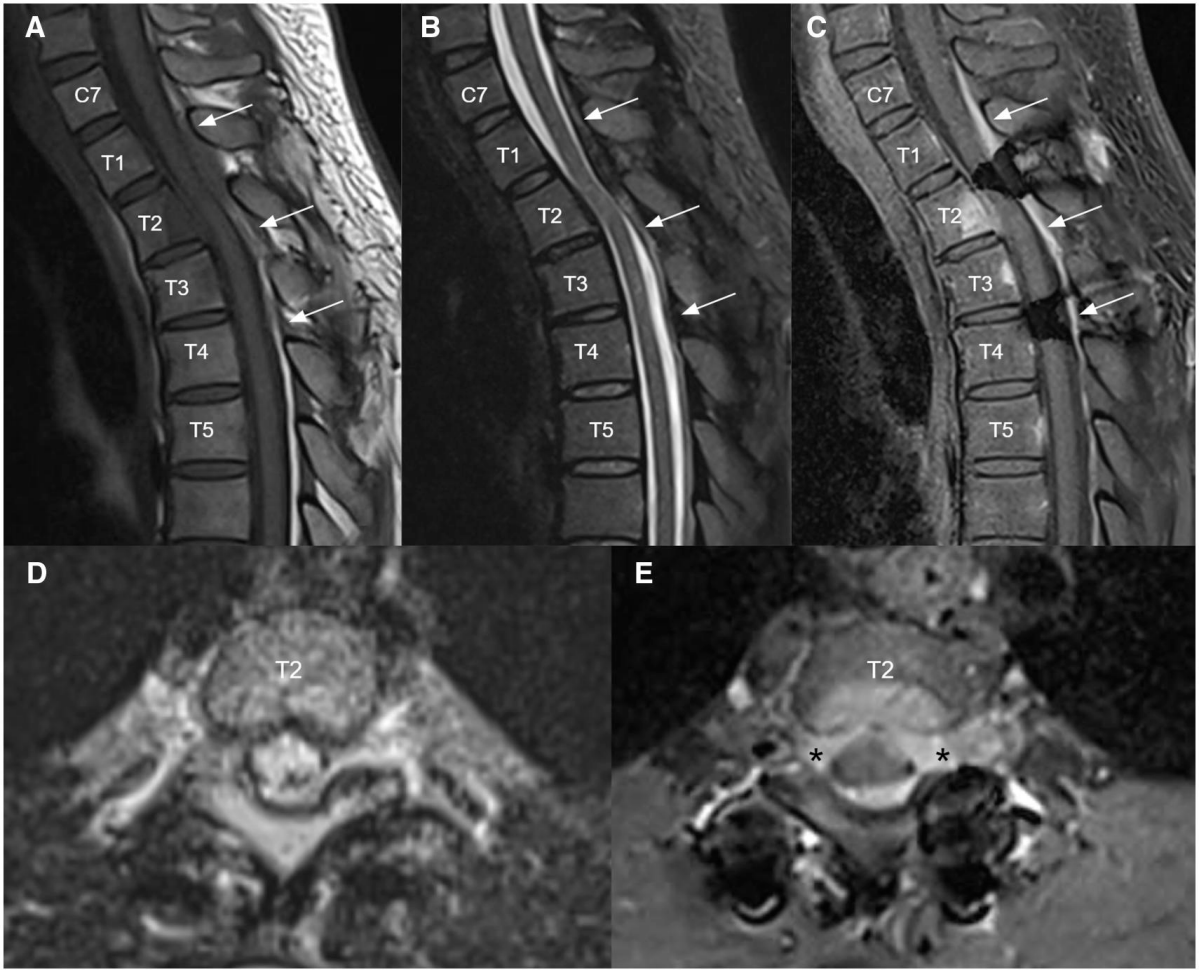
Follow up MRI 3 months after clinical recurrence upon low dose corticosteroid for a few years. Sagittal T1 weighted (A), T2 STIR (B), and T1 weighted post-contrast (C) MRI images of upper thoracic spine demonstrated a new enhancing infiltrative posterior epidural mass at C7 to T4 levels (white arrows) causing mild cord compression mainly at T2 level with cord edema. Axial T2 stir (D) and T1 weighted post-contrast (E) at T2 level showed that the epidural mass infiltrates into bilateral T2 lateral recesses and bilateral T2/3 intervertebral foramina (black asterisks). A new enhancing lesion was also noted at the posterior aspect of T2 vertebral body with overlying thin anterior epidural thickening (C, E). Mild residue T2 STIR hyperintense signal intensity (B) in the spinal cord at T3 level was compatible with old insult.

Further stepwise reduction of low dose corticosteroid to 4 mg daily over the next 4 years, and then recurrence of symptoms was noticed. MRI of the thoracic spine was repeated (Figure 4A-C) and demonstrated a new enhancing infiltrative posterior epidural mass at C7 to T4 levels causing mild cord compression mainly at T2 level with cord oedema. The epidural mass infiltrated into bilateral T2 lateral recesses and bilateral T2/3 intervertebral foramina (Figure 4D and E). A new enhancing lesion was also noted at the posterior aspect of the T2 vertebral body (Figure 4C and E). Mild residue T2/STIR hyperintense signal intensity (Figure 4B) in the spinal cord at T3 level was compatible with old insult. Patient’s symptoms responded well to higher dose of corticosteroid combined with disease-modifying antirheumatic drugs.

## Discussion

IgG4-RD was first reported in 2001 in Japan. It can affect different organs, mimicking malignancy, and infection.^2^ Common organs involved including salivary gland (chronic sclerosing sialadenitis), thyroid (Riedel thyroiditis), orbit (orbital pseudotumour), mediastinum (fibrosing mediastinitis), pancreas (autoimmune pancreatitis), biliary tree (sclerosing cholangitis), and retroperitoneum (retroperitoneal fibrosis).^1^^,^^2^ The clinical symptoms would depend on the organ affected.^1^^,^^2^ About 58%-88% of patients show multiple organ involvement, while the remainders are isolated events.^2^

The Japanese IgG4-RD team established the comprehensive diagnostic criteria for IgG4-RD in 2011 and the 2020 revised comprehensive diagnostic (RCD) criteria has been newly adopted, suggesting that the diagnosis of IgG4 requires a combination of clinical, haematological, and histopathologic evidence (Table 1).^2^

**Table 1.** The 2020 revised comprehensive diagnostic (RCD) criteria for IgG4-RD.^2^

1. Clinical and radiological features: One or more organs show diffuse or localized swelling or a mass or nodule characteristic of IgG4-RD. In single organ involvement, lymph node swelling is omitted. 2. Serological diagnosis: Serum IgG4 levels greater than 135 mg/dL. 3. Pathological diagnosis: Positivity for two of the following three criteria: a. Dense lymphocyte and plasma cell infiltration with fibrosis. b. Ratio of IgG4-positive plasma cells/IgG-positive cells greater than 40% and the number of IgG4-positive plasma cells greater than 10 per high powered field c. Typical tissue fibrosis, particularly storiform fibrosis, or obliterative phlebitis

Definite Diagnosis: 1 + 2 + 3; Probable Diagnosis: 1 + 3; Possible Diagnosis: 1 + 2.

Table 1 shows that elevation of serum IgG4 concentration is not a gold standard for IgG4-RD diagnosis and a cut off level of 135 mg/dL was adopted in the 2020 RCD criteria. Histopathologically, dense infiltration of lymphocyte and plasma cells with fibrosis is a characteristic of IgG4-RD. In almost all patients with IgG4-RD, the ratio of IgG4-positive plasma cells among IgG-positive cells is more than 40%.^2^ Since storiform fibrosis and obliterative phlebitis observed in haematoxylin and eosin staining are reported to be unique and characteristic features for IgG4-RD, they are helpful in diagnosis in the case of poor IgG and/or IgG4 staining.^2^

IgG4-HP is a rare form of IgG4-related disease manifestation. As in other types of IgG4-RD manifestations, it is more common in middle-aged or elderly males.^3^ There is limited literature in this disease entity because neurological involvement in IgG4-RD is relatively rare. Isolated manifestation in the spine as hypertrophic pachymeningitis is more unusual compared to intracranial involvement.^3^ The first case of IgG4-related spinal pachymeningitis with spinal cord compression was reported in 2009^5^ and there were less than 50 cases reported in the literature so far. A nationwide survey conducted in Japan revealed crude prevalence of HP was 0.949/100 000 population and only 8.8% were IgG4-HP.^6^ There was also a marked male predominance in the study with male to female ratio of 1:0.17.^6^ In a national case registry of IgG4-RD in France, the frequency of pachymeningitis was 4.1%.^7^ A previous literature review reported 37.8% of the spinal IgG4-HP cases were in the epidural space.^3^

IgG4-HP can present as a mass-like lesion on MRI. Patients often present with symptoms that result from mass effect or neurovascular compression.^4^ It is important to excluded other possible causes of epidural mass including infection (eg, neurosyphilis, bacterial meningitis, tuberculosis), malignancy (eg, lymphoma, metastasis) or other forms of inflammation (e,g, granulomatosis with polyangiitis, sarcoidosis). Organ-specific diagnostic criteria for central nervous system or pachymeningitis in IgG4-RD have not been established. As mentioned in previous literature, clinical history, serologic tests, cerebrospinal fluid studies, and imaging alone could not accurately identify the cause of hypertrophic pachymeningitis.^4^ Studies showed that serum IgG4 level are rarely informative and there is no specific CSF marker or well-established diagnostic value of CSF IgG4 oligoclonal band levels for IgG4-HP.^4^^,^^7^ Therefore, tissue biopsy with histopathologic studies is crucial for accurate diagnosis.^4^^,^^7^

Plaque-like dural thickening on CT can be very subtle. MRI is a better imaging modality to support the diagnosis, delineate the extent of disease, and follow up after treatment. It commonly shows T1 hypo-/iso-intense dural thickening with post-gadolinium homogeneous enhancement.^3^ The lesion can be markedly T2/FLAIR hypointense due to underlying fibrosis.^3^^,^^8^ 18F-FDG PET/CT is sensitive to active inflammation in IgG4, so it is useful in detecting systemic manifestations of IgG4-related disease and can be utilized to assess treatment response.^8^

IgG4-RD generally responds well to corticosteroids in its inflammatory stage, but relapse is not uncommon which is seen in 24%-54% of patients after reduction of corticosteroid dose in previous studies.^9^ Current treatment recommendations for IgG4-HP are tissue biopsy followed by high dose corticosteroid therapy and early decompressive surgery to avoid chronic neurological complications.^7^^,^^10^^,^^11^ Surgical decompression is necessary when there is progressive neurological symptoms caused by spinal cord compression.^3^ High-dose corticosteroids are generally effective while other immunosuppressive agents have shown variable efficacy in reducing the meningeal hypertrophy which may be used as a second-line treatment.^10^^,^^12^ Majority of patients presented with symptomatic relief and neurological recovery after initial treatment.^3^ However, long-term maintenance therapy with corticosteroid treatment and/or other immunosuppressive agents (the optimal treatment dose and has not been clarified in the literature) is essential to prevent recurrence.^3^

## Conclusion

This is a rare case of IgG4-RD manifesting as epidural mass, which mimics malignancy or infection on imaging. It is essential to include this alternative differential diagnosis for cases of idiopathic HP. Biopsy is necessary for accurate diagnosis. Recent consensus recommendations on initial treatment include early surgery and high-dose corticosteroids to avoid chronic neurological complications, followed by long-term corticosteroid maintenance therapy to prevent recurrence.

## Learning points

- Biopsy is essential for accurate diagnosis of IgG4 HP since serum IgG4 level is rarely informative.
- Radiologists and clinicians should consider IgG4-HP as a differential diagnosis in patients with an epidural spinal mass because prompt surgical and medical treatment may avoid long-term neurological complications.
